# Proposals for the classification of human rhinovirus species A, B and C into genotypically assigned types

**DOI:** 10.1099/vir.0.053686-0

**Published:** 2013-08

**Authors:** Chloe L. McIntyre, Nick J. Knowles, Peter Simmonds

**Affiliations:** 1 Roslin Institute, University of Edinburgh, Easter Bush, Edinburgh EH15 9RG, UK; 2 Pirbright Institute, Ash Road, Pirbright, Woking, Surrey GU24 0NF, UK

## Abstract

Human rhinoviruses (HRVs) frequently cause mild upper respiratory tract infections and more severe disease manifestations such as bronchiolitis and asthma exacerbations. HRV is classified into three species within the genus *Enterovirus* of the family *Picornaviridae*. HRV species A and B contain 75 and 25 serotypes identified by cross-neutralization assays, although the use of such assays for routine HRV typing is hampered by the large number of serotypes, replacement of virus isolation by molecular methods in HRV diagnosis and the poor or absent replication of HRV species C in cell culture. To address these problems, we propose an alternative, genotypic classification of HRV-based genetic relatedness analogous to that used for enteroviruses. Nucleotide distances between 384 complete VP1 sequences of currently assigned HRV (sero)types identified divergence thresholds of 13, 12 and 13 % for species A, B and C, respectively, that divided inter- and intra-type comparisons. These were paralleled by 10, 9.5 and 10 % thresholds in the larger dataset of >3800 VP4 region sequences. Assignments based on VP1 sequences led to minor revisions of existing type designations (such as the reclassification of serotype pairs, e.g. A8/A95 and A29/A44, as single serotypes) and the designation of new HRV types A101–106, B101–103 and C34–C51. A protocol for assignment and numbering of new HRV types using VP1 sequences and the restriction of VP4 sequence comparisons to type identification and provisional type assignments is proposed. Genotypic assignment and identification of HRV types will be of considerable value in the future investigation of type-associated differences in disease outcomes, transmission and epidemiology.

## Introduction

Human rhinoviruses (HRVs) are highly prevalent respiratory pathogens traditionally associated with mild and self-limited upper respiratory tract infections (‘colds’). The replacement of relatively insensitive virus isolation methods with molecular methods for HRV detection has contributed to a recent reappraisal of their incidence of infection and genetic diversity. HRVs are now known to be intimately linked to the development and exacerbations of respiratory diseases such as asthma (Khetsuriani *et al.*, 2008; Miller *et al.*, 2007) and responsible for more severe disease manifestations such as bronchiolitis in young children and in the immunosuppressed (Gutman *et al.*, 2007; Ison *et al.*, 2003; McErlean *et al.*, 2007). Genetic characterization of HRVs detected by molecular methods has revealed much greater diversity than previously described; approximately one-third of HRV infections are now known to be caused by a third group of rhinoviruses (species C), uncultureable *in vitro* and therefore entirely missed by traditional virus isolation methods (Arden *et al.*, 2006; Kaiser *et al.*, 2006; Kistler *et al.*, 2007a; Lamson *et al.*, 2006; Lau *et al.*, 2007; Lee *et al.*, 2007; McErlean *et al.*, 2007; Olenec *et al.*, 2010; Renwick *et al.*, 2007).

HRVs are members of the genus *Enterovirus* of the family *Picornaviridae*, a large virus group that additionally contains human enteroviruses and a number of viruses infecting non-human primates, pigs, cattle, sheep and other mammals (Knowles *et al.*, 2012). The genus *Enterovirus* has recently been divided into a total of 12 species based on their genetic divergence, sites of replication and disease associations (http://www.picornaviridae.com). Among these species, three are designated *Rhinovirus A*, *Rhinovirus B* and *Rhinovirus C*, a nomenclature that reflects their primary tropism for the respiratory tract. Currently, humans are the only known natural host, although it is possible that primates could also be susceptible. *Rhinovirus B* and most *Rhinovirus A* variants use the intracellular adhesion molecule-1 (major) receptor for cell entry (Uncapher *et al.*, 1991), whilst a subset of *Rhinovirus A* types use the low-density lipoprotein receptor (Hofer *et al.*, 1994). The receptor(s) used by HRV species C (HRV-C) types is currently unknown. HRVs are most commonly transmitted by the respiratory–salivary route, both by person-to-person contact and by airborne transmission. In temperate countries, infections occur primarily in two peaks, the first between April and May and the second between September and October (Monto, 2002; Vesa *et al.*, 2001).

HRVs within each species show remarkable genetic and, where determined, antigenic heterogeneity. Species A and B isolates were originally characterized serologically, with 75 and 25 serotypes being classified by cross-neutralization assays by 1987 (Hamparian *et al.*, 1987; Kapikian *et al.*, 1967, 1971), and prototype strains of each were lodged at the American Type Culture Collection (ATCC). However, as well as being laborious, time-consuming and requiring extensive panels of antisera specific to each serotype, classification by neutralization cannot be used for species C rhinoviruses because of their refractoriness to cell culture.

Human enteroviruses (EVs) are classified into four species (*Enterovirus A*, *Enterovirus B*, *Enterovirus C* and *Enterovirus D*) and, like HRVs, isolates have also been traditionally characterized by neutralization assays. Some neutralization determinants are located on the external VP1 protein in the virus nucleocapsid, and it has been shown that sequence divergence in this region correlates with serotype designation of poliovirus isolates (Kilpatrick *et al.*, 1998) and other enteroviruses (Kiang *et al.*, 2009; Oberste *et al.*, 1999a, b). Indeed, a threshold of 25 % nucleotide and 12 % amino acid sequence divergence generally corresponds to the previous serological division of EVs into members of the same or different serotypes (Oberste *et al.*, 1999a). As well as providing a simpler method for EV type identification than the previously used serological assays, VP1 sequencing is now routinely used for identification and assignment of new EV types without serological confirmation of antigenic differences (Brown *et al.*, 2009; Oberste *et al.*, 2002, 2004, 2007).

In the current study, we investigated whether a comparable threshold of sequence diversity in the VP1 of HRVs would enable a similar genotypic division of HRV-A, -B and -C variants into types and additionally whether this correlated with sequence divergence in the VP4/VP2 region (routinely used for HRV typing). This investigation was prompted by the increasing use of molecular methods to characterize HRVs and the consequent lack of isolates or antisera with which to antigenically characterize them and, secondly, by the difficulty in culturing species C HRVs required for neutralization assays (Bochkov *et al.*, 2011; Kistler *et al.*, 2007a; McErlean *et al.*, 2007).

This investigation follows on from previously published proposals for the classification of HRV-C based on a 13 % divergence threshold in VP1 (Simmonds *et al.*, 2010). These have been adopted by the *Picornaviridae* Study Group as the primary mechanism for defining HRV-C types. We have extended this classification system for species A and B rhinoviruses. The previously used term ‘serotype’ reflects the identification and classification of HRV variants by antigenic properties and therefore assignment by genetic comparison methods requires a different or combined term. Following the terminology now used for enteroviruses, we have adopted the term ‘type’ throughout the study to represent those HRV variants that have been identified and classified by either cross-neutralization or genetic comparisons.

## Results

### Assigning nucleotide divergence thresholds in the VP1 region of HRV-A, -B and -C

**Fig. 1.**
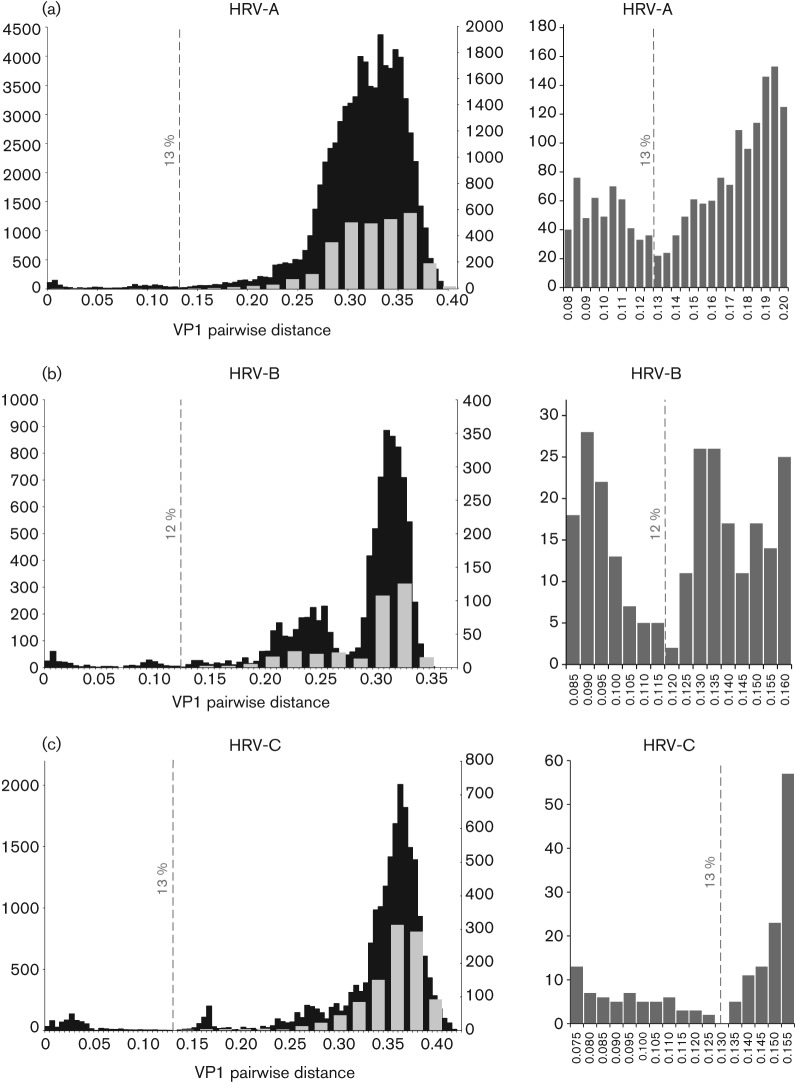
Distributions of pairwise nucleotide *p*-distances for the VP1 region of HRV-A (a), -B (b) and -C (c). Left graphs show number of sequences of the pairwise distances between all available VP1 sequences in black (left *y*-axis) and a comparison of prototype strains only in grey (right *y*-axis). The proposed thresholds for type assignment are shown as dotted lines. Right graphs show the sequences distances around the proposed assignment thresholds for each species shown using an expanded *x*-axis scale.

In order to determine whether a distinct threshold that divided pairwise *p*-distance comparisons in the VP1 region into intra- and inter-type values existed within each HRV species, distributions of pairwise nucleotide *p*-distances were constructed from a total of 435 HRV-A, 133 HRV-B and 206 HRV-C VP1 sequences (Fig. 1; sequences used for species C represent an expanded dataset from the one analysed previously; Simmonds *et al.*, 2010). These showed a maximum within-species nucleotide *p*-distance of 39.4, 33.9 and 42.9 % for species A, B and C, respectively, and minimum values of between 10 and 14 % divergence. Detailed inspection allowed identification of minimum values in the distribution and assignment of a nucleotide divergence threshold for each species (Fig. 1, right-hand panels). A threshold of 13 % was identified for both HRV-A and -C sequences, whilst the less-divergent HRV-B isolates showed a minimum value that supported a 12 % threshold.

**Fig. 2.**
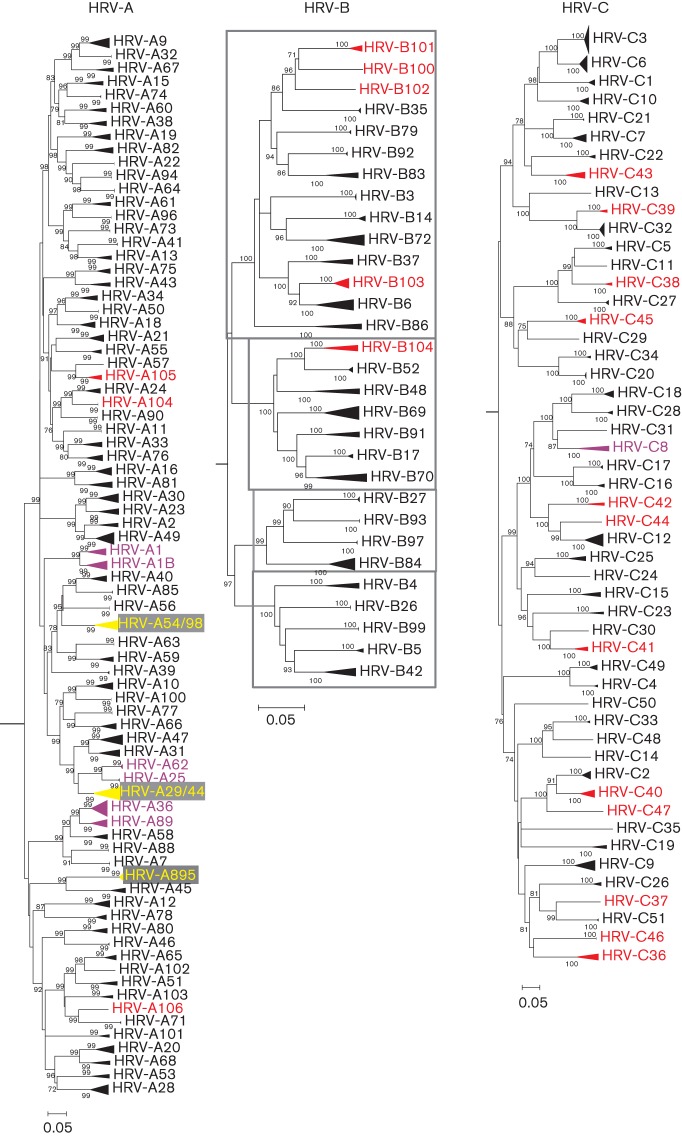
Neighbour-joining phylogenetic trees showing the VP1 region of HRV-A, -B and -C. The tree was constructed from by a neighbour-joining method from 100 bootstrap resampled sequence alignments of maximum composite likelihood distances. Multiple sequences of the same type are shown as triangles with heights proportional their numbers and depths corresponding to their earliest diverging branch. Within HRV-B, phylogenetic clusters with pairwise *p*-distances ranging from 0.1900 to 0.2600 are indicated by grey-outlined boxes. Instances where two types have been combined to form a single type are indicated in yellow. New HRV types defined on the basis of sequence divergence in VP1 are shown in red and HRV types with VP1 intra- or inter-type divergence outside the proposed thresholds are shown in purple. Bars, 0.05 nt substitutions per site.

The total datasets of available VP1 sequences contained unequal representations of different types within each species, ranging from one to a maximum of 13 for HRV-A (A49), 10 for HRV-B (B69) and 22 for HRV-C (C3). Particularly for pairwise distance comparisons, these may potentially distort the shape of the distribution of inter-type distances because comparisons with certain types are over-represented in the distance totals. An additional analysis was therefore carried out with only single sequences from each type (grey shading superimposed in Fig. 1, left graphs). For each, prototype strains of HRV-A and -B were those of the first full-genome sequence of a deposited ATCC isolate that was deposited in GenBank (highlighted in red in Table S1 in JGV Online). Prototype strains of HRV-C were those listed previously (Simmonds *et al.*, 2010; Table S1). This closely reproduced the analysis of the whole VP1 dataset in the shape and range of the inter-type distribution of distances (Fig. 1). Whilst distance distributions between VP1 sequences of HRV-A and -C showed a single peak, those of HRV-B showed a bimodal distribution. The peak between *p*-distance values of 0.19 and 0.26 corresponded to comparisons between HRV-B types within each of the four basally branching phylogenetic clusters (indicated by grey-outlined boxes in Fig. 2). The larger peak thus corresponded to comparisons between variants in different clusters.

### Phylogenetic analysis of the VP1 region

Phylogenetic trees were constructed of the VP1 region of HRV-A, -B and -C (Fig. 2). All HRV sequences fell into bootstrap-supported monophyletic groups that closely matched types assigned by sequence distances. These groups were numbered either by clustering with a known prototype strain (HRV-A/B 1–100), by order of submission of full-genome (HRV-A101–106, HRV-B100–104 and HRV-C1–11) or VP1 sequences (HRV-C12–51). The vast majority of sequences within each terminal clade showed intra-clade VP1 divergence substantially less than the proposed thresholds of 13 % in HRV-A and -C and 12 % in HRV-B (Table 1). Most of these also showed a minimum inter-clade VP1 divergence (with a different assigned type) that was much greater than the proposed threshold.

**Table 1.** Limits of intra- and inter- clade VP1 *p*-distance for HRV-A, -B and -C New HRV types defined on the basis of sequence divergence in VP1 are indicated in bold.

Type	Intra*	Inter†
**A1**	**0.0906**	**0.1185**
**A1B**	**0.1092**	**0.1185**
A2	0.1060	0.1407
A7	0.0046	0.1816
**A8**	**0.0035**	**0.0153**
A9	0.1085	0.1476
A10	0.0999	0.1701
A11	0.0000	0.1722
A12	0.0999	0.2625
A13	0.0816	0.1713
A15	0.1034	0.1638
A16	0.0889	0.1719
A18	0.0859	0.1754
A19	0.0977	0.2272
A20	0.0999	0.1504
A21	0.0952	0.2054
A22	0.1133	0.1903
A23	0.1072	0.1429
A24	0.0760	0.1310
**A25**	**0.0059**	**0.0934**
A28	0.1123	0.2280
**A29**‡	**0.0072**	**0.0724**
A30	0.1107	0.1429
A31	0.1166	0.1404
A32	0.0012	0.1476
A33	0.1026	0.1608
A34	0.0964	0.1580
**A36**	**0.1150**	**0.1200**
A38	0.0943	0.1916
A39	0.0094	0.2340
A40	0.1214	0.1597
A41	0.0057	0.1713
A43	0.1096	0.1606
**A44**‡	**0.0072**	**0.0724**
A45	0.0813	0.2521
A46	0.0057	0.2027
A47	0.1216	0.1404
A49	0.1001	0.1407
A50	0.0023	0.1580
A51	0.0991	0.1903
A53	0.1027	0.2220
**A54**	**0.0872**	**0.1072**
A55	0.0557	0.2066
A56	0.0000	0.2006
**A57**	**0.0000**	**0.1285**
A58	0.0862	0.1567
A59	0.0897	0.1362
A60	0.0977	0.1965
A61	0.1017	0.1804
**A62**	**0.0071**	**0.0934**
A63	0.0000	0.1737
A64	0.0058	0.1580
A65	0.0653	0.1903
A66	0.0845	0.1302
A67	0.0946	0.1778
A68	0.1044	0.1504
A71	0.0045	0.1719
A73	0.0034	0.1822
A74	0.0058	0.1638
A75	0.0785	0.1606
A76	0.0921	0.1608
A77	0.0012	0.1852
A78	0.1162	0.2625
A80	0.0871	0.2027
A81	0.1298	0.1719
A82	0.1050	0.1951
A85	0.0058	0.1597
A88	0.0011	0.1816
**A89**	**0.0879**	**0.1200**
A90	0.0000	0.1743
A94	0.0000	0.1580
**A95**	**0.0000**	**0.0153**
A96	0.0000	0.1804
**A98**	**0.0000**	**0.1072**
A100	0.0000	0.1468
A101	0.0611	0.2608
A102	0.0000	0.1509
A103	0.0685	0.2162
A104	0.0000	0.1310
**A105**	**0.0671**	**0.1285**
A106	0.0000	0.1719
B3	0.0045	0.2176
B4	0.0938	0.1957
B5	0.0139	0.1667
**B6**	**0.0881**	**0.1134**
B14	0.0178	0.1862
B17	0.0068	0.1242
B26	0.0022	0.1863
B27	0.0034	0.1762
B35	0.0134	0.1910
B37	0.0782	0.1474
B42	0.0984	0.1667
B48	0.1101	0.1876
B52	0.0057	0.1209
B69	0.1017	0.1810
B70	0.1160	0.1242
B72	0.0992	0.1862
B79	0.0046	0.1924
B83	0.0884	0.1695
B84	0.1013	0.2226
B86	0.1048	0.2184
B91	0.0846	0.1427
B92	0.0046	0.1695
B93	0.0024	0.1762
B97	0.0035	0.1937
B99	0.0012	0.1817
B100	0.0000	0.1655
B101	0.0157	0.1627
B102	0.0000	0.1627
**B103**	**0.0351**	**0.1134**
B104	0.0845	0.1209
C1	0.0515	0.2293
C2	0.0569	0.1650
C3	0.0388	0.1460
C4	0.0158	0.1394
C5	0.0421	0.1704
C6	0.0663	0.1460
C7	0.0751	0.1516
**C8**	**0.1315**	**0.2196**
C9	0.1103	0.2818
C10	0.0368	0.2049
C11	0.0000	0.1704
C12	0.1007	0.1965
C13	0.0045	0.2640
C14	0.0000	0.2147
C15	0.0917	0.2714
C16	0.0440	0.1473
C17	0.0175	0.1473
C18	0.0527	0.1925
C19	0.0724	0.3017
C20	0.0073	0.1510
C21	0.0098	0.1516
C22	0.0368	0.2500
C23	0.0813	0.2699
C24	0.0000	0.2274
C25	0.1088	0.2274
C26	0.0394	0.2611
C27	0.0308	0.2239
C28	0.0348	0.1925
C29	0.0000	0.2390
C30	0.0000	0.1842
C31	0.0000	0.2196
C32	0.0282	0.1357
C33	0.0209	0.1890
C34	0.0266	0.1510
C35	0.0000	0.3263
C36	0.1111	0.2564
C37	0.0000	0.2069
C38	0.0344	0.1735
C39	0.0257	0.1357
C40	0.0702	0.1650
C41	0.0748	0.1842
C42	0.0856	0.2407
C43	0.1157	0.2466
C44	0.0000	0.1965
C45	0.0428	0.2390
C46	0.0000	0.2564
C47	0.0000	0.2217
C48	0.0000	0.1890
C49	0.0311	0.1394
C50	0.0000	0.2859
C51	0.0049	0.2069

* Maximum VP1 *p*-distance observed within a bootstrap-supported phylogenetic clade or putative type.

† Minimum VP1 *p*-distance observed between one particular bootstrap-supported phylogenetic clade and its nearest neighbour.

‡ In cases where contemporary strains group separately from prototype strains of two types, which should potentially be combined (A29/A44), the *p*-distance listed involves the prototype strains only.

### Identification of new types on the basis of sequence divergence in VP1

Consistent with the thresholds identified for each species, three new types of HRV-A and three of HRV-B have been proposed previously; HRV-A101 (Rathe *et al.*, 2010), HRV-A102 (de Vries *et al.*, 2008), HRV-A103, HRV-B100 (Linsuwanon *et al.*, 2011), HRV-B101 and HRV-B102 (McIntyre *et al.*, 2013) (http://www.picornaviridae.com). In addition, the current analysis provided evidence for three further new HRV-A types (tentatively labelled HRV-A104–A106) and two novel HRV-B types (HRV-B103 and -B104), labelled in red in Fig. 2. The additional sequence data provided in this study and published elsewhere for HRV-C now create a phylogenetic tree that can be divided into 51 distinct clades. These have been assigned as types based on sequence divergence in the VP1 region (Table 1), adding a further 18 types to the total of 33 first defined in 2010 (Simmonds *et al.*, 2010) (http://www.picornaviridae.com).

Although several of the putative new HRV-A and -B types included showed minimum VP1 distances far greater than the assignment thresholds shown in Fig. 1, several were close to or below the 13 and 12 % levels (Table 1). As examples of the former, proposed new HRV-B types B100, B101 and B102 showed minimum pairwise *p*-distances in VP1 of 0.214, 0.190 and 0.227 from their nearest confirmed HRV-B type (HRV-B35). Pairwise nucleotide *p*-distances between these three new HRV-B types ranged from 0.163 to 0.171. All proposed species C types were similarly clearly above the assignment threshold of 13 % for this species (Table 1). However, other variants proposed as new types showed minimum distances that were much closer to the threshold; for example, the six available sequences from the novel group putatively designated HRV-B103 showed VP1 distances from their nearest neighbour type (HRV-B6) ranging from 0.113 to 0.148. However, for these two types, only two pairwise comparisons fell below the 12 % threshold and both involved sequence F110_9318.

The newly designated HRV-B104 represented a division of a previously single type, HRV-B52, based on the existence of two discrete phylogenetic clades with VP1 *p*-distances of 0.121–0.142 between them. In this case, the clade containing the original ATCC isolates, including strains FJ445188 (GenBank accession no.) and EF173424, has retained the HRV-B52 designation, whereas the clade containing field isolates FJ445137 and JF781506 has been reassigned as HRV-B104. Similar variability was observed in species A where two of the proposed new types showed distances close to the assignment threshold (HRV-A104 and -A105).

### Reassignment of types with pairwise distances below assignment thresholds

**Fig. 3.**
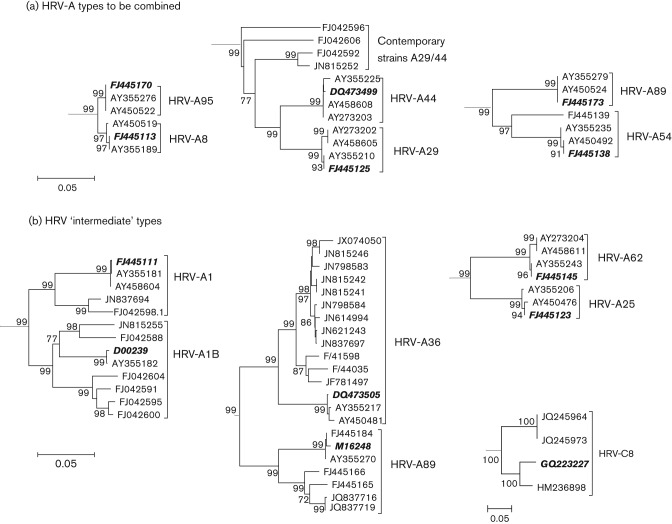
Neighbour-joining phylogenetic trees of HRV-A type pairs that do not conform to the proposed VP1 divergence thresholds. Sequences of previously recognized prototype isolates of each HRV type are highlighted in bold and italic. (a) Three HRV-A type pairs that have been combined. (b) Four HRV type pairs that displayed intermediate divergence values are shown. Bars, 0.05 nt substitutions per site.

Analysis of pairwise VP1 divergence between previously recognized HRV-A types also revealed several examples of VP1 distances below the 13 % threshold. For example, HRV-A8 and HRV-A95 showed an inter-type VP1 *p*-distance range of 0.015–0.019. We propose that all sequences of these two types are reassigned as HRV-A8, a proposal consistent with their previously documented serological cross-reactivity (Ledford *et al.*, 2004) (Fig. 3a).

Similarly, HRV-A29 and HRV-A44 have previously demonstrated cross-reactivity (Cooney *et al.*, 1982) and prototype strains showed an inter-type VP1 *p*-distance of 0.0724 (Fig. 3a). We propose that these sequences be reassigned as HRV-A29. Interestingly, contemporary sequences that belonged to HRV-A29 by VP1 *p*-distance from the prototype strains (maximum *p*-distance of 0.127) showed a maximum divergence of 0.159 when compared with each other. All contemporary HRV-A29 sequences grouped separately on phylogenetic trees from both the HRV-A29 and HRV-A44 prototype sequences (Fig. 3a).

HRV-A54 and HRV-A98 are a third type pair that are more closely related than expected, with a pairwise nucleotide VP1 *p*-distance of 0.107–0.124 (Fig. 3a). Although cross-neutralization properties of these two types have not been analysed in previous studies (Cooney *et al.*, 1982; Ledford *et al.*, 2004), we propose that all variants classified as these two types are reassigned as HRV-A54.

In addition to those type pairs that have been combined into single type assignments, a number of other HRV types displayed intermediate divergence values that violated the proposed thresholds. These included HRV-A1A and -A1B, which are serologically cross-reactive (Cooney *et al.*, 1982) but showed several pairwise *p*-distances that fell above the VP1 threshold (Fig. 3). We propose that members of these two clades retain their assignment as HRV-A1, although this should be reviewed as more sequences and confirmatory serological data become available. As a contrasting problem, the type pair HRV-A25 and -A62 showed VP1 pairwise distances ranging from 0.093 to 0.103 from each other (Fig. 3b), below the assignment threshold, despite showing no cross-neutralization (Cooney *et al.*, 1982). We propose leaving these assignments unchanged pending confirmation of the serological relationship between these two types. Similarly, prototype isolates of types HRV-A36 and -A89 (GenBank accession nos DQ473508 and M162488) showed VP1 *p*-distances of 0.1292, just below the assignment threshold (Fig. 3b). For these types, the HRV-A36 antiserum can neutralize HRV-A89 (as well as HRV-A50 and HRV-A58). However, the antiserum to HRV-A89 cannot neutralize HRV-A36 and almost all pairwise distances between more recent isolates of HRV-A36 (*n* = 9) and HRV-A89 (*n* = 5) are above the assignment threshold. We propose to retain their current designations as HRV-A36 and HRV-A89.

Only one HRV-C type showed *p*-distance values that violated proposed thresholds. HRV-C8 strains were divided into two clades, one previously classified as HRV-Cpat28 and both containing contemporary sequences (Fig. 3b). Although the VP1 *p*-distance between the two clades ranged from 0.129 to 0.132, we propose that these two clades should remain classified as HRV-C8 on the basis of borderline VP1 divergence.

**Fig. 4.**
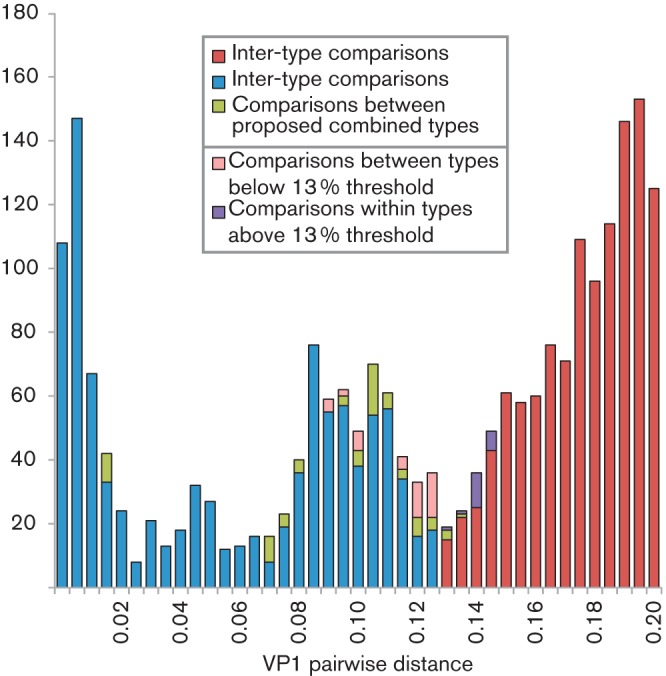
Distribution of inter- and intra-type HRV-A pairwise *p*-distances around the proposed 13 % VP1 divergence threshold. VP1 *p*-distances between or within types that are consistent with the proposed 13 % type-assignment threshold for VP1 are shown in blue (intra-type) and red (inter-type). Pairwise distances between types that we propose should be combined are shown in green. Comparisons that violate the VP1 assignment threshold are shown in pink (different HRV types but with distances falling below the threshold) and purple (intra-type comparisons falling above the divergence threshold). The *y*-axis shows the number of sequences.

Despite these examples, the number of pairwise distances in the overall distribution that were close to the assignment threshold (and therefore potentially ambiguously assigned) was small (Fig. 4). The majority represented comparisons between type pairs that should be combined (Fig. 4, green). Only six HRV-A types showed any between-type distances that were below the threshold (pink), and there were three with within-type distances that were above (Fig. 4, purple). As described above, their current classification can incorporate phylogenetic data and any available information on neutralization properties in addition to VP1 distances. Combined, these may potentially resolve any remaining uncertainties over type assignments.

At the conclusion of this analysis of all available HRV VP1 sequences, totals of 77 HRV-A types, 29 HRV-B and 51 HRV-C types have been listed in full in Tables S1 and S2). Novel strains of HRV were classified based on date of submission to the *Picornaviridae* Study Group, rather than the earliest isolated strain.

### HRV classification using VP4/2 sequences

**Fig. 5.**
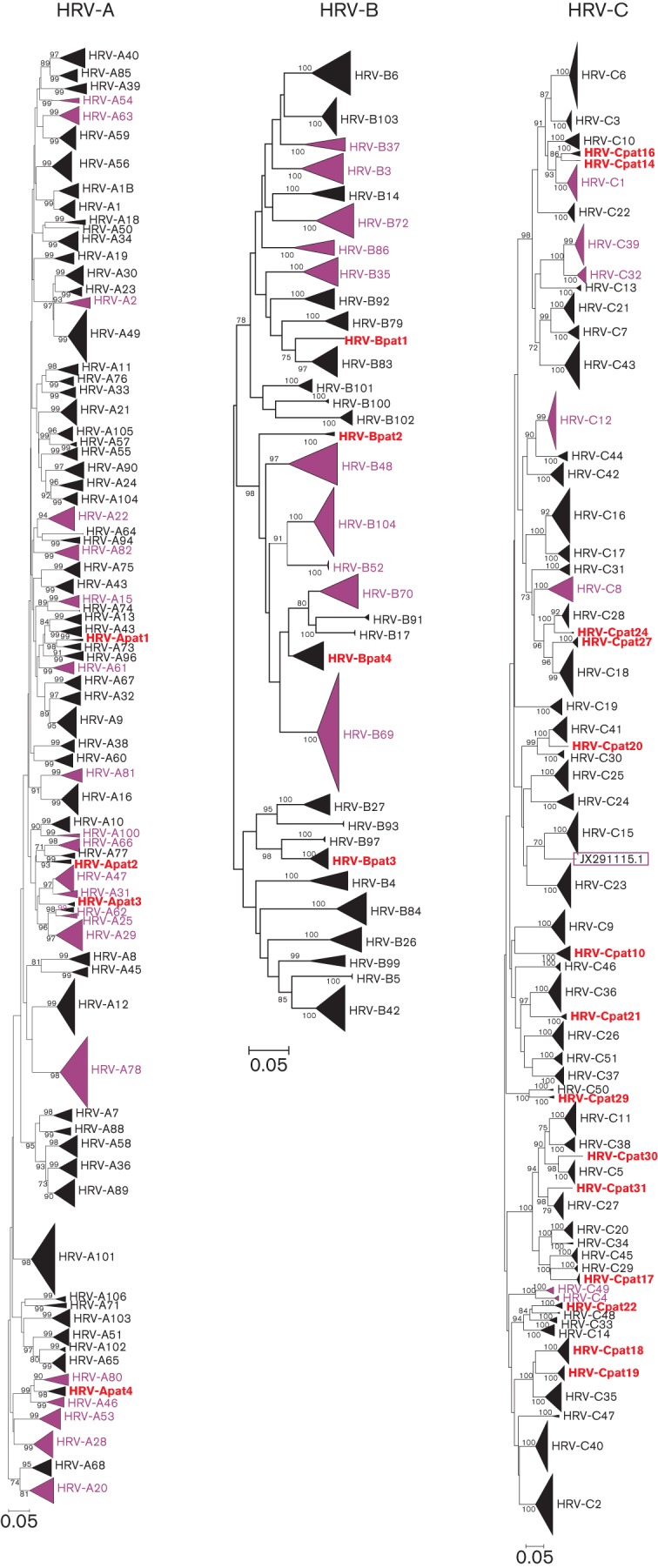
Phylogenetic analysis of all available VP4/VP2 sequences of HRV-A, -B and -C. The tree was constructed as described for Fig. 2. Multiple sequences of the same type have been depicted as in Fig. 2. Groups that have been temporarily designated provisionally assigned types (PATs) are shown in red. HRV types that do not conform to the proposed VP4/VP2 thresholds are shown in purple. The single recombinant sequence detected, JX291115, is indicated by a purple box. Bars, 0.05 nt substitutions per site.

The well-conserved and readily amplified VP4/VP2 region of HRV is commonly used in studies of its epidemiology and clinical associations, with over 3900 HRV sequences from this region in GenBank, over 10 times the number of VP1 sequences (*n* = 384). To investigate the value of VP4/VP2 sequences for HRV type identification, we performed phylogenetic analysis on the entire dataset and compared groupings of sequences of known type to those observed in VP1 (Fig. 5).

The majority of HRV strains grouped congruently between VP1 and VP4/VP2. Only one sequence showed evidence of phylogenetic incongruence; JX291115 is a member of HRV-C51 type in the VP1 region, with a pairwise nucleotide *p*-distance of 0.01 to JF317015 (HRV-C51 prototype strain). However, in VP4/VP2, this sequence showed a VP1 *p*-distance of 0.146 from JF317015 and lay closest to HRV-C15. Its status as a recombinant sequence was demonstrated by triplet analysis using the rdp software package. In contrast, comparable analysis of all other HRV-C and HRV-A/B variants with both full VP4/2 and VP1 sequences available by rdp and gard (164 types in species A, 70 in species B and 145 in species C) demonstrated no evidence of recombination between regions.

**Fig. 6.**
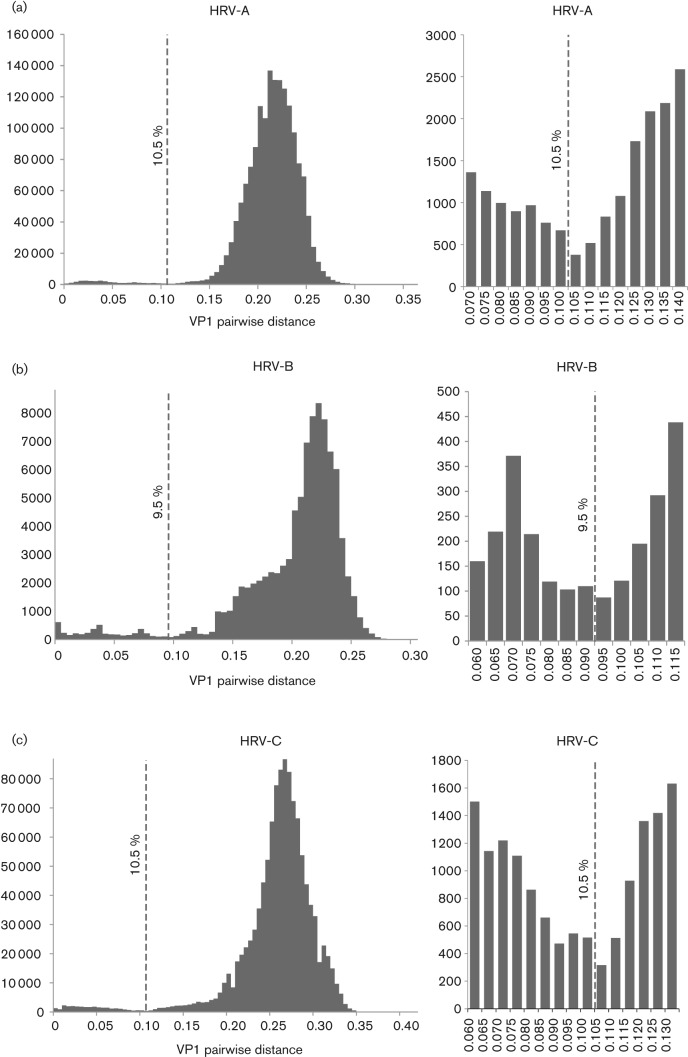
Distributions of pairwise nucleotide *p*-distances for the VP4/VP2 region of HRV-A, -B and -C. The figure is labelled as in Fig. 1.

We previously proposed a divergence threshold of 10 % for identifying different HRV-C types using VP4/VP2 sequences (Simmonds *et al.*, 2010). Using the current much larger dataset of HRV-C sequences and a shorter fragment (positions 616–1004, based on typical coordinates of sequences available in GenBank), pairwise *p*-distance distributions were plotted, along with those for species A and B (Fig. 6). Detailed inspection of distributions of pairwise distances showed possible assignment thresholds of 10.5, 9.5 and 10.5 % for HRV-A, -B and -C, respectively, based on minimum values. The shorter length and greater sequence conservation than VP1 resulted in less-well-defined thresholds and a greater proportion of pairwise comparisons close to the assignment threshold, which may cause problems with type identification.

For example, four type pairs showed VP4/VP2 divergence that was below the proposed threshold [HRV-A31/A47 (0.083), HRV-B52/B104 (0.093), HRV-C4/C49 (0.100) and HRV-C32/C39 (0.090)] even though they were previously assigned as different types based on VP1 divergence and separate clustering by phylogenetic analysis (Table 1 and Fig. 2). More problematic was the larger number of HRV variants of the same type with VP4/VP2 divergence above the assigned thresholds (22, 10 and six types of HRV-A, -B and -C, respectively; Table S3). Most of these types contained several contemporary sequences that had diversified substantially from their corresponding prototype sequence. In these cases, *p*-distances above the threshold occurred between contemporary isolates diversifying on separate evolutionary lineages, while retaining similarity in the intra-type range with their corresponding prototype type strain [VP4/VP2 distances of <10.5 % (species A) or 9.5 % (species B)]. This is similar to the pattern observed within the VP1 region of contemporary strains of HRV-A29.

### Using VP4/VP2 sequences for assignment of provisionally assigned types (PATs)

The original HRV-C classification included 28 PATs, which were temporarily assigned on the basis of VP4/VP2 sequence divergence alone (Simmonds *et al.*, 2010) until a matching VP1 sequence was obtained. Of these original 28 PATS, 16 have since been reassigned as new (confirmed) types based on VP1 divergence (new HRV-C type designations listed in Table S1 and S2), whilst one was combined into an existing type (HRV-C8/Cpat28; see above). VP1 sequences are unavailable for the remaining 11 PATs (Table S4).

Eight further divergent VP4/VP2 groups were identified among species A (*n* = 4), and species B (*n* = 4) (Table S4). A further three provisionally assigned HRV-C types were also detected (designated HRV-Cpat29-pat31). All showed divergence above the VP4/VP2 threshold proposed for their species. These await sequence data from VP1 to confirm their assignment as different types.

### Geographical distribution of HRV types

**Fig. 7.**
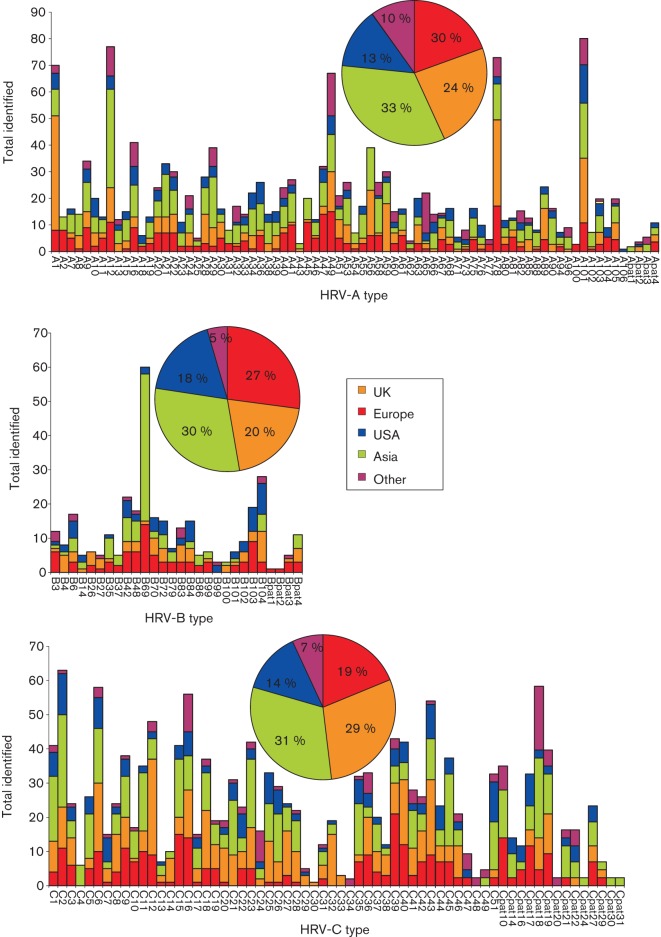
Detection frequencies of different HRV species and types reported from different geographical locations. Total numbers of sequences from each geographical area are shown in the inset pie chart. This analysis excluded HRV types for which geographical information was limited or unavailable (HRV-A50, -A57, -A64, -B5, -B17, -B91 and -C50).

The distribution of HRV types (identified using the larger dataset of VP4/VP2 sequences and excluding PATs) was compared for the UK, the rest of Europe, the USA, Asia and other regions (Australia, the Middle East and Africa). Each geographical zone contained similar relative frequencies of HRV-A, -B and -C (Fig. 7). Individual types showed considerable variability in detection frequency, although those that were common showed similar detection frequencies in different geographical areas. Similarly, none of the prevalent HRV types showed any geographical restriction, findings that suggest that most or all HRV types circulate freely worldwide.

## Discussion

### Genotypic identification of HRV species and types

The ability to perform genotyping directly from clinical specimens removes the need for cell culture passage and therefore greatly reduces the time required for type identification for all three HRV species. It additionally allows variants refractory to virus isolation, such as species C, to be identified. This parallels the transition from isolation and neutralization assay to VP1 sequencing for the identification of EV (sero)types (McWilliam Leitch *et al.*, 2009; Mirand *et al.*, 2009; Nix *et al.*, 2006). Because of its shorter length, greater sequence conservation and the possibility of using the same set of primers for amplification of all three HRV species, the VP4/VP2 region has been most commonly used for genetic characterization of HRV in clinical samples (Blomqvist *et al.*, 2009; Han *et al.*, 2009; Mizuta *et al.*, 2010; Savolainen-Kopra *et al.*, 2009; Wisdom *et al.*, 2009a). Comparison of its predictive value compared with type assignments based on VP1 sequences provides evidence for its suitability of HRV type identification along with some restrictions. The latter include uncertainties in type identifications of variants showing sequence divergences from other HRV sequences close to the threshold assigned to that species. Examples of both inter-type distances below this threshold and intra-type distances above have been presented (Table S3), although patterns of phylogenetic clustering of variants provide further contributory information for type identification.

Overall, however, the thresholds we have proposed divide HRV sequences into groups that correspond most consistently with phylogenetic groupings (with the small number of exceptions we have specifically itemised). Placing thresholds in any other position in the distance distributions would create groupings that conflict with those defined by phylogenetic analysis and by serology relationships. Placing the threshold at a minimum point in the distribution additionally minimizes the number of conflicting between- and within-group distances within an assigned type.

The identification and definition of HRV types by using genetic data within the capsid region has the potential to revolutionize HRV typing. The ease with which this can be carried out, compared with traditional virus culture and neutralization assays, should make the endeavour of classifying all detected HRV sequences much more feasible and attractive. A more extensive global or sentinel system of type identification should allow large-scale investigations of HRV epidemiology, transmission and evolution; sequence data for comparisons are available worldwide almost instantly and will be of considerable value in surveillance for the emergence of new types of species with differing clinical presentations from those traditionally attributed to HRV.

### Proposed criteria for the division of HRV into genotypically assigned types

In addition to type identification, the definition of robust nucleotide divergence thresholds for the definition of new EV types (25 %) now provides the primary means for the assignment of new EV types (Oberste *et al.*, 1999a, b). This has been used for several years and has created the assignment of 36 new EV types numbered from EV-73 onwards (Brown *et al.*, 2009; Norder *et al.*, 2003; Oberste *et al.*, 2004, 2007; Smura *et al.*, 2007a, b; Tapparel *et al.*, 2009; Yozwiak *et al.*, 2010). We propose an equivalent system for the assignment of new HRV types in which criteria similar to those proposed for the assignment of species C rhinoviruses (Simmonds *et al.*, 2010) are extended to HRV-A and -B. HRV does, however, differ from EVs in showing lower divergence thresholds for type assignments (12–13 % nucleotide sequence divergence in VP1) and a lack of capsid recombination that enables VP4/VP2 sequences to be used at least for type identification. The criteria for HRV have been formulated as follows:

1. An HRV type should be phylogenetically distinct from other HRV types.
2. Analysis of divergence in the VP1 region should include at least 90 % of the full VP1 coding region for each variant.
3. In the VP1 region, an HRV type should have at least 13 % (HRV-A), 12 % (HRV-B) or 13 % (HRV-C) nucleotide divergence from all other HRV types.
4. In cases where minimum nucleotide divergence in VP1 of a putative new type lies close to the threshold for that species (±1 %), patterns of phylogenetic grouping with existing types may contribute to its assignment as would any available information on serological cross-reactivity.
5. VP4/VP2 sequences are predictive of HRV species and type and can be used for type identification in epidemiological studies.
6. HRV variants divergent from all known VP4/VP2 sequences (i.e. above type-assignment thresholds for the identified species in this region) but without an available VP1 sequence may be designated a PAT.
7. Subsequent determination of VP1 sequences for PATs would allow their reassignment as new types if they show VP1 sequence divergence above the threshold for that species).
8. New HRV type names should be numbered sequentially and separately for each species – the next assignments for the three rhinovirus species would be HRV-A107, HRV-B105 and HRV-C52.
9. The ‘prototype strain’ of each new HRV type should be the first reported full-genome sequence or, if this is unavailable, the first reported full VP1 sequence.
10. The *Picornaviridae* Study Group should continue to oversee assignment of new HRV types, as the group includes members with specific expertise in EV type assignment and a number of scientists currently active in HRV research.

The use of only capsid-coding regions in type-assignment criteria should not detract from the importance of continued investigation of other genomic regions, in particular where these may contribute to the phenotype and disease associations of HRV. In addition, the use of the VP1 region for definition of new HRV types should not discourage the widespread continued use of 5′-untranslated region and VP4/VP2 screening protocols. Screening with these relatively conserved regions allows a much greater opportunity for discovery of previously unknown HRV types through further sequence characterization of VP1 and other genome regions. Although this represents, to our knowledge, the most comprehensive survey of HRV sequence data currently possible, the guidelines should be subject to further review as additional data becomes available.

In all three species, the phylogeny of the full VP1 region reliably separated isolates of known differing type into distinct bootstrap-supported clades. This is consistent with the presence of a large number of the neutralizing immunogenic sites in HRV-A and -B (Kistler *et al.*, 2007b) that underlie their serological properties. The difficulties with culturing species C rhinoviruses have to date prevented their serological properties from being directly investigated. It is possible that the sinus mucosal organ culture system used to culture HRV-C15 might prove more broadly applicable for other species C variants (Bochkov *et al.*, 2011). Limited serological reinvestigation of a number of previous HRV-A and -B type assignments would be useful for isolates where cross-reactivity is uncertain (such as HRV-A54/A98) or conflicts with their sequence divergence in VP1.

There is a possibility that a smaller fragment of VP1 could be suitable for genotyping in HRV. Oberste *et al.* (1999b) reported that a 450 nt segment at the 3′ end of VP1 was effective for EV type identification and the results had a 100 % correlation with neutralization results. A second study showed full correlation of a 303 nt stretch of VP1 with neutralization data, but this was only applied to 59 strains (Kiang *et al.*, 2009). In addition, a simple, fast and effective method of typing EVs by pyrosequencing has recently been developed (Silva *et al.*, 2008). This may become extremely useful as pyrosequencing becomes more widely available worldwide. Whether a shorter region than the whole VP1 would be similarly predictive of HRV types and could be used for future type assignments requires further investigation.

## Methods

### 

#### Selection of samples.

VP4/VP2 sequences generated from several studies of HRV molecular epidemiology in the UK (Gaunt *et al.*, 2011; Harvala *et al.*, 2012; McIntyre *et al.*, 2010, 2013; Simmonds *et al.*, 2010; Wisdom *et al.*, 2009a, b) (Simmonds *et al.*, unpublished data) (431 HRV-A and 113 HRV-B) were divided into groups based on phylogenetic clustering. Any isolate that did not show a close grouping with a prototype full genome or represented a distinct phylogenetic cluster of HRV-C was selected for amplification of the VP1 region. The procedure for amplification of the VP1 region of HRV-A, -B and -C and initial descriptions of sequences generated have been described previously (McIntyre *et al.*, 2010, 2013; Simmonds *et al.*, 2010).

#### Sequence alignment, calculation of pairwise nucleotide *p*-distances and phylogenetic analysis.

All available HRV VP4/VP2 and VP1 sequences were downloaded from GenBank in November 2012. The genome regions analysed in this study included the full VP1 region and a fragment of VP4/VP2 (nt 616–1004 numbered according to GenBank accession no. EF582385 for HRV-C4) that is commonly used in studies of HRV epidemiology (Blomqvist *et al.*, 2009; Han *et al.*, 2009; Henquell *et al.*, 2012; Kaida *et al.*, 2011; Miller *et al.*, 2009; Mizuta *et al.*, 2010). Any sequences that were <90 % complete across these regions or wereclassified as non-functional were excluded. All sequences were aligned using the sse v1.0 sequence editor package (Simmonds, 2012) and pairwise nucleotide *p*-distances were calculated using the program Sequence Distances within the package. Phylogenetic trees were constructed in the mega v5.0 software package (Tamura *et al.*, 2011) by the neighbour-joining method (Saitou & Nei, 1987) from 100 bootstrap resampled sequence alignments of maximum composite likelihood (Guindon & Gascuel, 2003) distances, with pairwise deletion for missing data. All phylogenetic analysis was undertaken using the *Enterovirus D* sequence (GenBank accession no. D00820 for EV-D70) as an outgroup.

#### Analysis of recombination within the capsid region of HRV-A, -B and -C.

Phylogenetic trees were inspected visually for evidence of incongruence between the VP4/VP2 and VP1 regions. Datasets containing these two non-consecutive regions were concatenated to give one continuous sequence, which was then additionally analysed for the occurrence of recombination. rdp v4.0 (Martin *et al.*, 2010) analysis was used to screen each HRV species dataset for the occurrence of recombination within the capsid-coding region. Each sequence set was examined using a combination of algorithms implemented in the software package, specifically rdp (Martin & Rybicki, 2000), GeneConv (Padidam *et al.*, 1999), MaxChi (Smith, 1992), Chimaera (Posada & Crandall, 2001), SiScan (Gibbs *et al.*, 2000) and Bootscan (Martin *et al.*, 2005). A threshold *P* value of <0.05 and detection by at least two algorithms was required for a recombination event to be detected. Each HRV species dataset was further analysed using the gard program (Kosakovsky Pond *et al.*, 2006) as part of the HyPhy package. Any predicted recombination breakpoints were analysed by construction of neighbour-joining maximum composite likelihood trees for sequence fragments 5′ and 3′ to the putative breakpoint.
